# Association between malnutrition-inflammation score (MIS) and quality of life in elderly hemodyalisis patients

**DOI:** 10.1590/2175-8239-JBN-2023-0171en

**Published:** 2024-09-16

**Authors:** Kelly Cristiane Rocha Lemos, Anália Nusya de Medeiros Garcia, Thais Oliveira Claizoni dos Santos, Nathalia Fidelis Lins Vieira, Ana Célia Oliveira dos Santos

**Affiliations:** 1 Universidade de Pernambuco, Faculdade de Ciências Médicas, Recife, PE, Brazil.; 2 Universidade de Pernambuco, Instituto de Ciências Biológicas, Recife, PE, Brazil.

**Keywords:** Malnutrition, Dialysis, Chronic Kidney Insufficiency, Elderly, Quality of Life

## Abstract

**Introduction:**

The malnutrition-inflammation process is one of the main causes of morbidity and mortality in patients with chronic kidney disease (CKD), influencing quality of life. The aim of this study was to identify the inflammatory and nutritional status of elderly hemodialysis (HD) and its association with quality of life.

**Methods:**

This study was carried out in health services in three different cities. The Malnutrition-Inflammation Score (MIS) was used to assess the inflammatory and nutritional status, with anthropometric measurements, protein status, lean mass and function. The quality of life was assessed using KDQOL-SF^TM^. Data were analyzed using multivariate analysis and the Poisson model to evaluate the factors that increased the risk of developing malnutrition and inflammation.

**Results:**

The MIS identified a 52.2% prevalence of malnutrition and inflammation in the population. In univariate analysis, most KDQOL-SF^TM^ domains presented higher scores for nourished elderly. Anthropometric measures associated with muscle mass and functionality were lower in the malnourished elderly. Multivariate modeling revealed a higher nutritional risk of 50.6% for women and older age, since with each additional year of life the risk of malnutrition increased by 2.4% and by 0.4% with each additional month on HD. Greater arm muscle circumference (AMC) and higher serum albumin were factors for reducing malnutrition by 4.6% and 34.7%, respectively.

**Conclusion:**

Higher serum albumin and preserved AMC have been shown to be good indicators of better nutritional status. Higher MIS was associated with poorer quality of life, older age, lower income and education, longer time on dialysis, and presence of comorbidities.

## Introduction

The number of people with chronic kidney disease (CKD) on maintenance hemodialysis (HD) has increased significantly in recent decades, mainly due to the aging of the population and the increase in the prevalence of hypertension and diabetes. On the other hand, dialysis therapy has improved, increasing the survival of patients in chronic dialysis programs^1,2^. The latest census by the Brazilian Society of Nephrology showed a total number of 153,831 patients on dialysis^3^.

Although dialysis techniques have continuously advanced, the mortality rate among patients with CKD remains high. The malnutrition-inflammation process is one of the main causes of morbidity and mortality in patients with CKD. This finding is illustrated by the inverse association between mortality rates and markers of adequate nutritional status^4^. Protein-energy wasting (PEW) is a condition of malnutrition, inflammation, anorexia and wasting of body reserves due to inflammatory and non-inflammatory conditions in CKD patients^5,6^.

The nutritional assessment of individuals on HD remains a significant challenge since there is no single method capable of accurately diagnosing the nutritional status of these patients^7^. The Malnutrition-Inflammation Score (MIS) is a practical and reproducible measure that can be used in both HD and peritoneal dialysis patients. Studies have shown that the MIS is associated with inflammation, anemia, quality of life and mortality. Higher MIS values are independently associated with a higher risk of hospitalization^8,9^.

While the association between quality of life (QoL) and MIS in CKD has been addressed in a number of studies, there is a lack of studies regarding this association in older patients. It should be highlighted that health-related QoL is a strong and independent predictor of hospitalization and death in patients undergoing dialysis^10^, and regular assessment of QoL is recommended as one of the parameters for adequacy of treatment by the Kidney Disease Outcomes Quality Initiative (K/DOQI)^11,12^.

The aim of this study was to identify the inflammatory and nutritional status of elderly hemodialysis patients and the association between this and their quality of life.

## Methods

This was a cross-sectional study conducted at three hemodialysis clinics in the metropolitan region of Recife in northeastern Brazil. The procedures complied with the ethical standards of the responsible committee on human experimentation and the Helsinki Declaration of 1975, as revised in 2013, and the protocol was approved by the Ethics Committee (CAAE 64859716.5.0000.5207). The informed consent form was signed by all participants. The sample consisted of all patients from the three nephrology clinics, recruited from March to December 2017, of both sexes, aged ≥ 60 years, who had undergone hemodialysis three times a week for at least six months. Elderly patients were excluded if they presented neoplasms, positive serology for human immunodeficiency virus (HIV), viral hepatitis, Alzheimer’s disease, cognitive impairment, amputated limb and/or wheelchair-bound, Parkinson’s disease and stroke sequelae.

**Figure 1:**
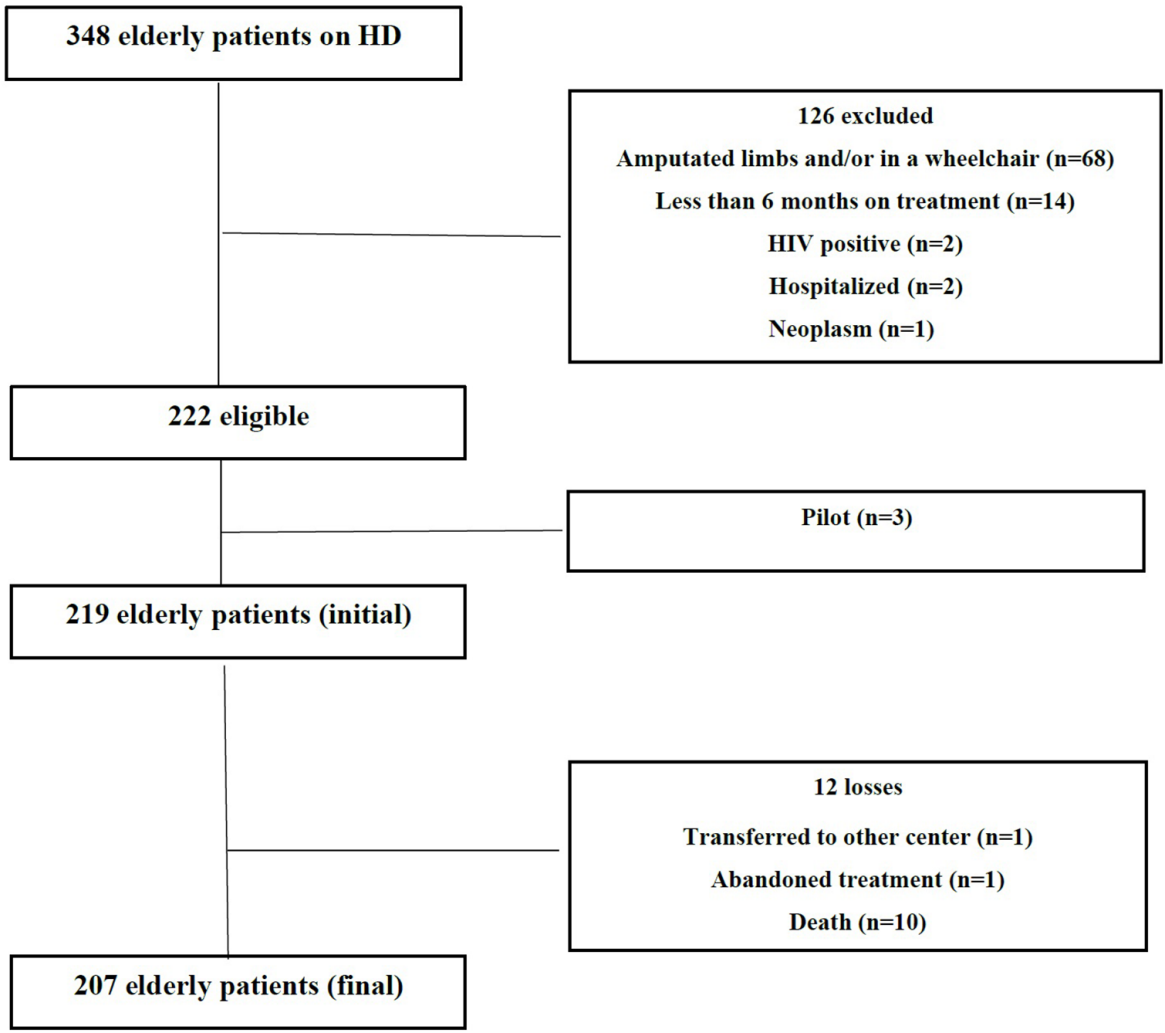
Sample selection protocol.

The sample selection protocol is described in Figure 1. Initially, a screening test for cognitive impairment, the Mini-Mental State Examination (MMSE), was carried out to identify which elderly people were eligible to take part in the study, with cut-off points of 18 and 23, depending on the participants’ level of schooling^13^.

Clinical, sociodemographic, and dialysis data were collected from clinical records and patients were questioned.

To assess patient perception of QoL, the KDQOL-SF™ questionnaire was used^14^.

Anthropometric measurements were performed after the HD session by three researchers all previously trained in nutrition tracking tools. Weight was measured on a Marte^®^ electronic scale. Height was measured with a portable Sanny^®^ stadiometer. To calculate the body mass index (BMI)^15^, the current weight (kg) was divided by the squared height (m), with the result expressed in kg/m^2^.

The arm circumference (AC) was measured on the non-vascular access arm using a Cescorf^®^ inelastic tape measure. The measurement was recorded in centimeters^16^.

To obtain the arm muscle circumference (AMC), measurements of the triceps skinfold (TS) on the non-vascular access arm and AC were entered into the Frisancho equation^16^.

To measure the TS, a Cescorf^®^ adipometer was used. The adequacy of the AMC was obtained through the 50^th^ percentile value (Frisancho^17^) and calculated by the Blackburn et al.^18^ equation, whereby a reduction of >10% was classified as being malnourished^19^.

Calf circumference (CC) was assessed on the left leg at the widest point and classified as malnourished with a CC <31 cm^20^.

A handgrip strength (HGS) test was performed on the patient’s hand with no arteriovenous fistula and was obtained using a Saehan^®^ hand dynamometer with a malnutrition cut-off of <23.3 kg specific to the hemodialysis population^21^.

Malnutrition-inflammation was assessed by the MIS, as recommended by Kalantar-Zadeh et al.^22^, and patients were classified as nourished when they scored less than 6 and malnourished with scores higher than or equal to 6^23^.

KDQOL – SF^TM^ according to the MIS score comprise specific and generic domains. The specific domains are List of symptoms/problems, Effects of kidney disease, Burden of kidney disease, Professional role, Cognitive Function, Quality of Social Interaction, Sexual Function, Sleep, Social support, Stimulation from dialysis team, and Patient Satisfaction. The generic domains are Physical Functioning, Role-Physical, Pain, General Health, Emotional well-being, Role-Emotional, Social Function, Energy/Fatigue^22^.

Laboratory parameters were collected from medical records according to the patient’s routine follow-up, during which a blood sample is collected before the hemodialysis session. The study considered the levels of phosphorus, calcium, parathyroid hormone, vitamin D, hemoglobin, and serum albumin.

Data were analyzed using SPSS. The sample description was performed by absolute and relative frequencies, means and standard deviations or medians and interquartile ranges of the assessed variables. For the bivariate analysis, the Chi-square association test, Student’s t test or Mann-Whitney test were used. In cases where the Chi-square test assumptions were not met, Fisher’s exact test was applied.

The multivariate analysis included factors with a significance of up to 20% in the bivariate analysis. The Poisson model with a robust variance was applied to assess the factors that increase the risk of developing malnutrition in the studied elderly patients. Factors with a significance level of 5% were maintained in the model. Moreover, the confidence intervals for the prevalence ratio and the Wald test were calculated to compare the risks between the levels of the assessed factors. All conclusions were drawn based on a significance level of 5%.

## Results

This study included 207 individuals, of which 131 were males (63.3%), aged between 60 and 94 (68.23 ± 6.68). Clinical and nutritional characteristics are described in Table 1, with results stratified by the MIS as nourished and malnourished.

**Table 1:** Clinical and nutritional characteristics according to the malnutrition-inflammation score (MIS) of older patients undergoing hemodialysis in northeastern brazil, (N = 207)

		Malnutrition-inflammation score (MIS)		
Characteristics	Total*	Nourished (MIS < 6)*	Malnourished (MIS = 6)*	p-value
	(n = 207)	(n = 99)	(n = 108)	
**Age** (years)**	68.23 ± 6.68	66.80 ± 4.96	69.55 ± 7.72	**0. 002** ^1^
Sex				
Female	76 (36.7)	24 (31.6)	52 (68.4)	**< 0. 001** ^3^
Schooling				
< 9 years (low schooling)	113 (54.6)	55 (48.7)	58 (51.3)	0.789^3^
Civil status (with partner)				
No partner	73 (35.3)	28 (38.4)	45 (61.6)	**0.044** ^3^
**Family income**				
< 1 minimum wage	4 (1.9)	2 (50)	2 (50)	0.921^3^
1–5 minimum wages	188 (90.8)	89 (47.3)	99 (52.7)	
> 5 minimum wages	15 (7.2)	8 (53.3)	7(46.7)	
**Nutritional Parameters**				
BMI (kg/m^2^)**	24.29 ± 4.21	25.22 ± 3.94	23.43 ± 4.28	**0.002** ^1^
AC (cm)**	28.10 ± 3.82	29 ± 3.32	27.29 ± 4.07	**0.001** ^1^
AMC (cm)**	23.82 ± 2.91	24.66 ± 2.47	23.04 ± 3.08	**< 0.001** ^1^
CC (cm)**	32.47 ± 3.38	33.43 ± 3.13	31.59 ± 3.38	**< 0.001** ^1^
HGS (kg)**	24.29 ± 8.73	27.10 ± 8.82	21.71 ± 7.85	**<0.001** ^1^
Serum albumin (g/dL)**	3.8 ± 0.33	3.86 ± 0.30	3.74 ± 0.34	**0.008** ^1^
**Comorbidities**				
Diabetes mellitus	100 (48.3)	47 (47)	53 (53)	0.818^3^
Systemic arterial hypertension	180 (87)	84 (46.7)	96 (53.3)	0.389^3^
Anemia	97 (46.9)	41 (42.3)	56 (57.7)	0.163^3^
**Dialysis Parameters**				
Dialysis dose (Kt/V)**	1.58 ± 0.36	1.52 ± 0.32	1.64 ± 0.38	**0.014** ^1^
Phosphorous (mg/dL)**	4.84 ± 1.11	4.91 ± 1.08	4.77 ± 1.15	0.373^1^
Calcium (mg/dL)**	8.87 ± 0.66	8.93 ± 0.68	8.82 ± 0.63	0.246^1^
Calcium-phosphorus product (mg^2^/dL^2^)**	42.29 ± 10.89	43.27 ± 10.57	41.39 ± 11.14	0.214^1^
PTH (pg/ mL)***	311.30 (168; 493)	338.10 (173; 519)	298.25 (165.25; 460)	0.454^2^
Vitamin D (ng/mL)**	31.88 ± 12.35	33.20 ± 13.11	30.68 ± 11.53	0.144^1^
Hemoglobin (g/100 ml)**	10.98 ± 1.4	11.09 ± 1.45	10.87 ± 1.45	0.271^1^
Time on HD (months)***	51 (22; 88)	43 (21; 78)	59 (24.50; 97.5)	**0.041** ^2^
Vascular access for HD				
*AVF or prosthetic*	154 (74.4)	74 (48.1)	80 (51.9)	0.912^3^
*Permcath or DLC*	53 (25.6)	25 (47.2)	28 (52.8)	
Length of HD session				
*3 to 3.5 hours*	8 (3.9)	4 (50)	4 (50)	1.000^4^
*4 hours*	199 (96.1)	99 (47.8)	108 (52.2)	

Abbreviations: BMI, body mass index; AC, arm circumference; AMC, arm muscle circumference; CC, calf circumference; HGS, handgrip strength; PTH, parathyroid hormone; HD, hemodialysis; AVF, arteriovenous fistula; DLC, double lumen catheter.

Notes: *Absolute and relative frequency for categorical variables;

**Mean ± SD;

***Median and interquartile range;

^1^p-value of Student’s t-test;

^2^p-value of Mann-Whitney test;

^3^p-value of Chi-square test;

^4^p-value of Fisher’s exact test.

The malnutrition-inflammation prevalence was 52.2% based on the MIS, and the average QoL score was 65.06 in the population studied based on the KDQOL-SF^TM^.

MIS was associated with age, female gender, and not having a partner. Most of the elderly patients had low schooling, a monthly income between 1 and 5 minimum wages, and their therapy was funded by the Brazilian Public Healthcare System (SUS). However, there was no difference in these characteristics between MIS categories.

In terms of the nutritional parameters, the mean BMI, AC, AMC, CC, HGS, and serum albumin were significantly lower in patients classified as malnourished by the MIS. There was a prevalence of malnutrition ranging from 54.1% by the HGS to 30.9% by the CC (Table 2). The comorbidities were similar between the groups.

**Table 2:** Prevalence of malnutrition according to each nutritional indicator in elderly patients undergoing hemodialysis in northeastern brazil (N = 207)

Nutritional indicator	Prevalence of malnutrition (%)
MIS (= 6)	52.2
Serum albumin (< 3.8 g/dL)	38.2
Reduced AMC (> 10%)	39.1
HGS (< 23.3 kg)	54.1
CC (< 31 cm)	30.9

bbreviations: MIS, Malnutrition-inflammation score; AMC, arm muscle circumference; HGS, handgrip strength; CC, calf circumference.

The dialysis parameters revealed that most of the elderly had an arteriovenous fistula (AVF)/prosthesis (74.4%) as a vascular access for hemodialysis and had 4-hour dialysis sessions (96.1%). The dialysis dose, determined by the Kt/V, presented better mean values in the malnourished group (1.64 ± 0.38 versus 1.52 ± 0.32, p = 0.014). The elderly who were most affected by the malnutrition-inflammation process were those who had been on dialysis for longer (43 versus 59 months, p = 0.041).

Table 3 describes the 19 domains of the KDQOL-SF^TM^ questionnaire. Most domains presented higher scores for the nourished elderly patients. In the specific domains List of Symptoms/Problems (p < 0. 001), Effects of kidney disease (p = 0.004), Burden of kidney disease (p = 0.004), Cognitive function (p = 0.001), Sleep (p = 0.002), Patient social support (p = 0.045), and satisfaction (p = 0.005), the QoL of the malnourished patients was significantly lower than those who were nourished.

**Table 3:** Scores of the KDQOL - SF^TM^ domains according to malnutrition-inflammation score (MIS) of elderly patients undergoing hemodialysis in northeastern brazil (n = 207)

		Malnutrition-Inflammation Score (MIS)		
Assessed dominion	Total**	Nourished (MIS < 6)**	Malnourished (MIS = 6)**	p-value
Specifics	(n = 207)	(n = 99)	(n = 108)	
List of symptoms/problems	79.22 ± 15.14	83.69 ± 13.62	75.13 ± 15.36	**< 0. 001** ^1^
Effects of kidney disease	69.38 ± 17.85	73.13 ± 16.40	65.94 ± 18.50	**0.004** ^1^
Burden of kidney disease***	50 (37.5; 75)	50 (43.75; 75)	50 (25; 62.5)	**0.004** ^2^
Professional role***	0 (0; 50)	0 (0; 50)	0 (0; 50)	0.413^2^
Cognitive Function	85.31 ± 17.08	89.56 ± 16.11	81.41 ± 17.07	**0.001** ^1^
Quality of Social Interaction	80.97 ± 17.38	83.29 ± 17.85	78.82 ± 16.72	0.064^1^
Sexual Function*	86.17 ± 22.31	91.53 ± 15.60	75.78 ± 29.39	0.059^1^
Sleep	69.24 ± 21.32	73.98 ± 20.39	64.88 ± 21.30	**0.002** ^1^
Social support	80.27 ± 28.23	84.34 ± 24.49	76.54 ± 30.91	**0.045** ^1^
Stimulation from dialysis team	72.89 ± 27.72	76.13 ± 26.06	69.90 ± 28.95	0.106^1^
Patient Satisfaction	64.65 ± 22.46	69.19 ± 21.86	60.49 ± 22.27	**0.005** ^1^
**Generic (SF)**				
Physical Functioning***	45 (25; 75)	60 (35; 85)	35(20; 58.75)	**<0.001** ^2^
Role-Physical***	50 (25; 100)	75 (50; 100)	25 (0; 75)	**<0.001** ^2^
Pain***	77.50 (42.50; 100)	80 (45; 100)	75.0 (32; 50)	**0.004** ^2^
General Health***	50(40; 70)	55 (45; 70)	50 (35; 65)	0.070^2^
Emotional well-being	72.37 ± 22.74	76.52 ± 23.27	68.55 ± 21.65	**0.011** ^1^
Role-Emotional***	66.67 (0; 100)	100 (33.33; 100)	66.7 (0; 100)	**0.002** ^2^
Social Function	74.52 ± 23.94	80.05 ± 22.44	69.44 ± 24.24	**0.001** ^1^
Energy/Fatigue	62.08 ± 22.62	64.54 ± 23.86	59.81 ± 21.27	0.133^1^

Notes: *(n = 47);

**Mean ± SD;

***Median and interquartile range;

^1^p-value of Student’s t-test;

^2^p-value of the Mann-Whitney test. In the dimensions of the KDQOL - SF^TM^ the value 0 reflects the lowest QoL and the value 100 reflects the highest QoL.

The generic QoL domains Physical functioning, Physical function, Pain, Emotional well-being, Emotional function, and Social function were also significantly different in nourished and malnourished elderly.

Table 4 presents the Poisson model adjustment for malnutrition and inflammation. After the bivariate analysis to assess the individual influence of factors on the development of malnutrition and inflammation, the combined analysis resulted in the following significant factors: gender, age, AMC measurement, serum albumin level, and time on HD. It was also observed that the female group presented a 50.6% greater risk of developing higher (worse) malnutrition-inflammation scores compared to the male group. For age, it was observed that with an increase of one year of life, there was a 2.4% increase in the risk of developing higher malnutrition-inflammation scores. For the AMC and albumin, there was a reduction of 4.6% and 34.7%, respectively, in the risk of developing malnutrition-inflammation with an increase of 1 cm in the AMC and of 1 g/dL in albumin. In terms of time on HD, for each month that the patient underwent treatment, this risk increased by 0.4%.

**Table 4:** Poisson model adjustment for a higher malnutrition-inflammation score (MIS)

Assessed factor	PR	95%CI	p-value
Sex			
*Male*	1.000	–	–
*Female*	1.506	1.154 – 1.965	0.003
Age	1.024	1.009 – 1.040	0.002
AMC	0.954	0.913 – 0.998	0.042
Albumin	0.653	0.452 – 0.943	0.023
Time on HD (months)	1.004	1.002 – 1.006	<0.001

Abbreviations: AMC, arm muscle circumference; HD, hemodialysis; PR, Prevalence Ratio; CI, Confidence Interval.

## Discussion

Assessing the nutritional status of HD patients is critical, since malnutrition, especially PEW syndrome, is highly prevalent and contributes to increased morbidity and mortality in patients on chronic HD^5,24^. The MIS is considered a sensitive parameter for assessing malnutrition and inflammation in HD patients, and a predictor of mortality^22^, but its use in the elderly population has been poorly documented.

According to the MIS, the prevalence of malnutrition-inflammation in the studied population was 52.2%. A more advanced age was associated with higher MIS levels, a result also found in other studies^25^. Aging is a risk factor for malnutrition, with an increase of 2.4% in risk for each year of life in the multivariate model for this population. Physiological changes take place in old age, resulting from reduced energy needs and expenditure, referred to as the anorexia of aging. This physiological anorexia increases the risk of weight loss and malnutrition when an older person develops a physical or psychological illness^26^.

For older people, food stands for family, union, and quality of life. The fact that patients live with a partner and reside with their family may increase their care at home. The most malnourished elderly patients were those who did not have a partner (61.6%). CKD causes functional losses that compromise independence and autonomy, which occurs more often with older patients^27^.

Women are usually less prevalent in CKD studies. The true protective effects of female hormones on the progression of kidney disease remain unknown. On the other hand, when on dialysis, women present poorer clinical parameters, including anemia, nutrition, and quality of life^28^. In this study, while 36.7% of the sample were women, 68.4% were malnourished and at a 50.6% increased risk of developing higher MIS scores than men.

The anthropometric and dialysis parameters were worse in the malnourished patients, which reinforces the importance of gaining body weight, especially lean mass. This is because weight gain reflects in better functional and immunological conditions, greater independence, and lower morbidity and mortality related to malnutrition^29^.

Serum albumin is an important nutritional parameter in patients undergoing renal replacement therapy, with a level of 3.8 g/dL considered normal. The elderly patients in this study were able to achieve this goal. Pereira et al.^30^ in a large study of 1,679 patients investigated the impact of albumin on mortality after two years of hemodialysis and found that mortality was significantly higher in the albumin group below 3.8 g/dL. Szuck et al.^8^, who verified the ability of nutritional indicators to predict the risk of hospitalization in hemodialysis patients, found that only serum albumin was able to predict this risk, and that patients with <3.8g/dL presented a 2.47 times higher incidence than those with higher albumin levels.

For the population of this study, the AMC was a protective factor for malnutrition-inflammation in the multivariate model, as with a one-centimeter increase in the AMC there was a 4.6% reduction in the risk of a higher MIS.

Regarding the adequacy of dialysis assessed by the measurement of Kt/V, the current European recommendation is a Kt/V equal to or greater than 1.2^31^. Kt/V is very important in the assessment of nutritional status, since inadequate dialysis results in a uremic state, progressing to nausea, vomiting, and anorexia, with consequent impairment in food intake. The results of this study demonstrate that dialysis was appropriate for these elderly patients, with a mean Kt/V of 1.58. The malnourished elderly patients presented the highest Kt/V levels, demonstrating that malnutrition can occur even when dialysis is efficient. Malnutrition reduces body volume, making the elderly more susceptible to an increase in Kt/V. The literature indicates that longer periods on HD are associated with poor nutritional status and inflammation^32^. In the sample studied, the risk of malnutrition-inflammation increased 0.4% for each month of dialysis therapy.

With increased access to new dialysis therapies, such as high-volume hemodiafiltration (HDF), and improvements in dialysis quality, the nutritional status of elderly patients on chronic dialysis may improve. In the study by Maduell et al.^33^, patients were randomized to continuous dialysis or HDF. The results showed that the normalized protein catabolic rate, which is a parameter of nutritional status, was higher in patients randomized to HDF.

The negative association between the nutritional condition/protein-energy wasting (PEW) and QoL has been demonstrated and its importance highlighted by several studies, since dialysis treatment, despite prolonging survival, has a great impact on various aspects of patients’ lives^34,35,36^. To date, there is no single method, such as a gold standard, capable of diagnose PEW, so the use of various nutritional markers is recommended^36^. Importantly, an ideal nutritional indicator should be able to predict clinical outcomes and identify patients who are in need of receiving nutritional interventions^37^. Despite dialysis therapy, PEW is common in dialysis patients and is related to inflammation, associated comorbidities, a hypercatabolic state, and decreased intake and anorexia^38^.

This study observed a negative association between MIS and several domains of the KDQOL-SF^TM^, suggesting that the occurrence of malnutrition-inflammation may have negatively affected the QoL of the elderly patients in this hemodialysis population. A systematic review demonstrated that malnourished older adults were more likely to have a poorer quality of life (p < 0.001; OR 2.85; CI 2.20–3.70). The authors also noted that when considering the effect of nutritional support in intervention studies, there is a significant improvement in the QoL of these individuals in both physical and mental aspects^39^.

In terms of the KDQOL-SF^TM^, the domain that obtained the highest score was sexual function, although it was not statistically associated statistically associated with the MIS.. This domain assesses whether patients have engaged in sexual activity over the last four weeks and the extent to which there have been problems with sexual arousal and satisfaction. This result should be carefully analyzed as part of the sample reported having an active sex life. However, erectile dysfunction is very common in patients with chronic kidney failure due to hormonal, physical, neurological, and psychological changes^40^.

Other high-scoring domains (better QoL) associated with MIS were cognitive function and social support. The social support domain verifies the support received from family and friends, and the domain of quality of social interaction assesses the family and social relationships with the patient. These aspects can be highly valued by elderly patients, as they are highly dependent on the care that hemodialysis requires from family members. It is important to involve the family in the treatment by encouraging their participation and guiding them to cooperate with the healthcare team^10^. When elderly people on renal replacement therapy are in an environment with supportive family and social relationships, they generally accept their treatment and are motivated and grateful^41^.

The item cognitive function assesses the participant’s perception of difficulties in concentrating and thinking, the presence of mental confusion, and the delay in reacting to phenomena that have happened or have been spoken of. Patients with CKD are at risk of cognitive decline. Cognitive impairment may be present at any stage of kidney disease and is associated with an increased risk of death and lower adherence to treatment. Although the mechanisms leading to loss of cognitive function in CKD are not completely clear, the literature demonstrates that clinical conditions, such as the elimination rate of uremic toxins, may induce neuronal lesions^41^. Oliveira et al.^10^ reported a significant inverse correlation between QoL and number of missed treatments, revealing that the lower the QoL in this aspect, the higher the number of missed treatments, thereby representing a lower adherence to treatment.

Sleep was one of the domains with the lowest score, and malnutrition-inflammation was significantly associated, since in this population, nourished patients slept better than those who were malnourished. Poor sleep quality is not uncommon in hemodialysis patients, with a prevalence ranging from 41% to 83%. It is most commonly associated with women, older age, the presence of depression, cardiovascular disease, poor quality of dialysis therapy and a compromised health-related quality of life^42^.

The low QoL score as an effect of kidney disease demonstrates how the limitations of dialysis, such as a restricted fluid intake and diet, travel difficulties, dependence on health staff, personal appearance and other limitations, are an inconvenience to elderly patients. The low QoL reflects the disruption that kidney disease brings to the lives of these patients, particularly because of the time they spend on treatment^41^. The results observed for the professional role score are related to the fact that almost all older people are no longer of productive age and those who are still working at the start of dialysis therapy often retire.

With regard to the generic domains (SF-36), the elderly patients in this study presented higher scores in the domains involving mental aspects, and lower scores in the domains involving physical aspects. With the aging process, the limitations caused by hemodialysis treatment tend to increase, especially the physical limitations. However, the emotional side becomes stronger with increasing age. Older people are psychologically better able to cope with the demands of treatment, their expectations are more realistic and they are more able to adapt to their state of health^10,32^.

The limitations of the study are related to the cross-sectional design, in which the group was only assessed once. Therefore, causal relationships cannot be established, i.e. whether malnutrition-inflammation causes impaired QoL, or if impaired QoL causes a decrease in appetite, and thus malnutrition. Cross-sectional studies with elderly people are subject to survival bias, as the most serious participants may have died, i.e. the results observed were from a population of senior survivors. In addition, the lack of analysis of fat-free mass loss and food intake made a more accurate assessment of nutrition in elderly patients impossible.

In summary, the results showed that the malnutrition-inflammation score was associated with poorer QoL for elderly hemodialysis patients. Serum albumin and AMC were found to be protective factors for high MIS.

Furthermore, elderly people with longer periods of renal replacement therapy, more years of life and women are at greater risk of developing malnutrition-inflammation.

## Data Availability

The data underlying this article are available in the article and in its online supplementary material.

## References

[1] Bello AK, Levin A, Lunney M, Osman MA, Ye F, Ashuntantang G (2019). International Society of Nephrology.

[2] Zoccali C, Moissl U, Chazot C, Mallamaci F, Tripepi G, Arkossy O (2017). Chronic fluid overload and mortality in ESRD. J Am Soc Nephrol.

[3] Nerbass FB, Lima HN, Moura JA, Lugon JR, Sesso R (2024). Censo brasileiro de diálise 2022. J Bras Nefrol.

[4] Chávez Valencia V, Mejía Rodríguez O, Viveros Sandoval ME, Abraham Bermúdez J, Gutiérrez Castellanos S, Orizaga de la Cruz C, et al (2018). Prevalencia del síndrome complejo de malnutrición e inflamación y su correlación con las hormonas tiroideas en pacientes en hemodiálisis crônica. Nefrologia.

[5] Riella MC. (2013). Nutritional evaluation of patients receiving dialysis for the management of protein-energy wasting: what is old and what is new?. J Ren Nutr.

[6] Pereira GR, Matos JPS, Ruzany F, Santos SFF, D’Almeida Fo E, Fernandes MS (2014). Alterações precoces da albumina sérica: impacto sobre a mortalidade aos 2 anos em pacientes incidentes em hemodiálise. J Bras Nefrol.

[7] Lee HJ, Son YJ. (2021). Prevalence and associated factors of frailty and mortality in patients with end-stage renal disease undergoing hemodialysis: a systematic review and meta-analysis. Int J Environ Res Public Health.

[8] Szuck P, Führ LM, Garcia MF, Silva AT, Wazlawik E (2016). Associação entre indicadores nutricionais e risco de hospitalização em pacientes em hemodiálise. Rev Nutr.

[9] Lopes MB, Silva LF, Lopes GB, Penalva MA, Matos CM, Robinson BM (2017). Additional contribution of the malnutrition-inflammation score to predict mortality and patient-reported outcomes as compared with its components in a cohort of african descent hemodialysis patients. J Ren Nutr.

[10] Oliveira APB, Schmidt DB, Amatneeks TM, Santos JC, Cavallet LHR, Michel RB. (2016). Qualidade de vida de pacientes em hemodiálise e sua relação com mortalidade hospitalizações e má adesão ao tratamento. J Bras Nefrol.

[11] National Kidney Foundation (2000). Clinical practice guidelines for nutrition in chronic renal failure. Am J Kidney Dis.

[12] Dehesa-López E, Correa-Rotter R, Olvera-Castillo D, González-Parra C, Baizabal-Olarte R, Orozco-Vega R (2017). Transcultural adaptation and validation of the Mexican version of the kidney disease questionnaire KDQOL-SF36 version 1.3. Qual Life Res.

[13] Bertolucci PHF, Campacci SR, Brucki SMD, Juliano YO (1994). Mini-exame do estado mental em uma população geral: impacto da escolaridade. Arq Neuropsiquiatr.

[14] Duarte PS, Miyazaki MCOS, Ciconelli RM, Sesso R. (2003). Tradução e adaptação cultural do instrumento de avaliação de qualidade de vida para pacientes renais crônicos (KDQOL-SFTM). Rev Assoc Med Bras.

[15] World Health Organization (2008). Global database on Body Mass Index [Internet]..

[16] Frisancho AR (1974). Triceps skin fold and upper arm muscle size norms for assessment of nutritional status. Am J Clin Nutr.

[17] Frisancho AR. (1990). Anthropometric standards for the assessments of growth and nutritional status.. Michigan: University of Michigan.

[18] Blackburn GL, Bistrian BR, Maini BS, Schlamm HT, Smith MF (1977). Nutritional and metabolic assessment of the hospitalized patient. JPEN J Parenter Enteral Nutr.

[19] Fouque D, Kalantar-Zadeh K, Kopple J, Cano N, Chauveau P, Cuppari L (2008). A proposed nomenclature and diagnostic criteria for protein–energy wasting in acute and chronic kidney disease. Kidney Int.

[20] Chumlea WC, Guo S, Roche AF, Steinbaugh ML (1988). Prediction of body weight for the nonambulatory elderly from anthropometry. J Am Diet Assoc.

[21] Garcia MF, Wazlawik E, Moreno YMF, González-Chica DA (2013). Diagnostic accuracy of handgrip strength in the assessment of malnutrition in hemodialyzed patients. ESPEN J.

[22] Kalantar-Zadeh K, Kopple JD, Block G, Humphreys MH (2001). A malnutrition-inflammation score is correlated with morbidity and mortality in maintenance hemodialysis patients. Am J Kidney Dis.

[23] Yamada K, Furuya R, Takita T, Maruyama Y, Yamaguchi Y, Ohkawa S (2008). Simplified nutritional screening tools for patients on maintenance hemodialysis. Am J Clin Nutr.

[24] Martin-Alemañy G, Valdez-Ortiz R, Olvera-Soto G, Gomez-Guerrero I, Aguire-Esquivel G, Cantu-Quintanilla G (2016). The effects of resistance exercise and oral nutritional supplementation during hemo dialysison indicators of nutritional status and quality of life. Nephrol Dial Transplant.

[25] Hanna RM, Ghobry L, Wassef O, Rhee CM, Kalantar-Zadeh K (2020). A practical approach to nutrition, protein-energy wasting, sarcopenia, and cachexia in patients with chronic kidney disease. Blood Purif.

[26] Crogan NL (2017). Nutritional problems affecting older adults. Nurs Clin North Am.

[27] Hendriks FK, Kooman JP, van Loon LJC (2021). Dietary protein interventions to improve nutritional status in end-stage renal disease patients undergoing hemodialysis. Curr Opin Clin Nutr Metab Care.

[28] Piccoli GB, Alrukhaimi M, Liu ZH, Zakharova E, Levin A (2018). What we do and do not know about women and kidney diseases; questions unanswered and answers unquestioned: reflection on World Kidney Day and International Woman’s Day. Nephrol Dial Transplant.

[29] Broers NJH, Canaud B, Dekker MJE, van der Sande FM, Stuard S, Wabel P (2020). Three compartment bioimpedance spectroscopy in the nutritional assessment and the outcome of patients with advanced or end stage kidney disease: what have we learned so far?. Hemodial Int.

[30] Pereira GRM, Strogoff-de-Matos JP, Ruzany F, Santos SFF, Almeida FE, Vasconcelos MSF (2015). Alterações precoces da albumina sérica: impacto sobre a mortalidade aos 2 anos em pacientes incidentes em hemodiálise. J Bras Nefrol.

[31] Tattersall J, Farrington K, Gentile G, Kooman J, Macias Núñez JF, Nistor I (2018). Is Kt/V useful in elderly dialysis patients? Pro and Con arguments. Nephrol Dial Transplant.

[32] Lim HS, Kim HS, Kim JK, Park M, Choi SJ (2019). Nutritional status and dietary management according to hemodialysis duration. Clin Nutr Res.

[33] Maduell F, Moreso F, Pons M, Ramos R, Mora-Macià J, Carreras J (2013). High-efficiency postdilution online hemodiafiltration reduces all-cause mortality in hemodialysis patients. J Am Soc Nephrol.

[34] de Roij van Zuijdewijn CL, Grooteman MP, Bots ML, Blankestijn PJ, van den Dorpel MA, Nubé MJ (2016). Comparing tests assessing protein-energy wasting: relation with quality of life. J Ren Nutr.

[35] Mazairac AH, de Wit GA, Penne EL, van derWeerd NC, Grooteman MP, van den Dorpel MA (2011). Protein-energy nutritional status and kidney disease-specific quality of life in hemodialysis patients. J Ren Nutr.

[36] Aghakhani N, Samadzadeh S, Mafi TM, Rahbar N (2012). The impact of education on nutrition on the quality of life in patients on hemodialysis: a comparative study from teaching hospitals. Saudi J Kidney Dis Transpl.

[37] Arshad AR, Jamal S, Amanullah K (2020). Agreement between two nutritional assessment scores as markers of malnutrition in patients with end-stage renal disease. Cureus.

[38] Kaysen GA, Greene T, Larive B, Mehta RL, Lindsay RM, Depner TA (2012). The effect of frequent hemodialysis on nutrition and body composition: frequent Hemodialysis Network Trial. Kidney Int.

[39] Ferraz SF, Freitas ATVS, Vaz IMF, Campos MIVAM, Peixoto MRG, Pereira ERS (2015). Estado nutricional e ganho de peso interdialítico de pacientes com doença renal crônica em hemodiálise. J Bras Nefrol.

[40] Rasheed S, Woods RT (2013). Malnutrition and quality of life in older people: a systematic review and meta-analysis. Ageing Res Rev.

[41] Papadopoulou E, Varouktsi A, Lazaridis A, Boutari C, Doumas M (2015). Erectile dysfunction in chronic kidney disease: from pathophysiology to management. World J Nephrol.

[42] Ma SJ, Wang WJ, Tang M, Chen H, Ding F (2021). Mental health status and quality of life in patients with end-stage renal disease undergoing maintenance hemodialysis. Ann Palliat Med.

