# Supplementary material for: Association between malnutrition-inflammation score (MIS) and quality of life in elderly hemodyalisis patients
Source: J Bras Nefrol. 2024 Sep 16;46(4):e20230171. doi: 10.1590/2175-8239-JBN-2023-0171en (PMC11539957; doi:10.1590/2175-8239-JBN-2023-0171en)
Supplement: Translation

# Translation: Associação entre o escore de desnutrição-inflamação (MIS) e qualidade de vida em pacientes idosos em hemodiálise

**DOI:** 10.1590/2175-8239-JBN-2023-0171pt

**Authors:** Kelly Cristiane Rocha Lemos, Anália Nusya de Medeiros Garcia, Thais Oliveira Claizoni dos Santos, Nathalia Fidelis Lins Vieira, Ana Célia Oliveira dos Santos

**Affiliations:** 1 Universidade de Pernambuco, Faculdade de Ciências Médicas, Recife, PE, Brasil.; 2 Universidade de Pernambuco, Instituto de Ciências Biológicas, Recife, PE, Brasil.

**Keywords:** Desnutrição, Diálise, Insuficiência Renal Crônica, Idosos, Qualidade de Vida

## Abstract

**Introdução:**

O processo de desnutrição-inflamação é uma das principais causas de morbimortalidade em pacientes com DRC, influenciando a qualidade de vida. O objetivo deste estudo foi identificar o estado inflamatório e nutricional de idosos em hemodiálise (HD) e sua associação com qualidade de vida.

**Métodos:**

Estudo realizado em serviços de saúde de três cidades diferentes. O Escore de Desnutrição-Inflamação (MIS, por sua sigla em inglês) foi utilizado para avaliar estado inflamatório e nutricional, com medidas antropométricas, estado proteico, massa magra e funcionalidade. A qualidade de vida foi avaliada com KDQOL-SF^TM^. Os dados foram analisados utilizando análise multivariada e modelo de Poisson para avaliar fatores que aumentaram o risco de desenvolver desnutrição e inflamação.

**Resultados:**

O MIS identificou prevalência de 52,2% de desnutrição e inflamação na população. Na análise univariada, a maioria dos domínios do KDQOL-SF^TM^ apresentou escores maiores para idosos nutridos. Medidas antropométricas associadas à massa muscular e funcionalidade foram menores em idosos desnutridos. A modelagem multivariada revelou maior risco nutricional de 50,6% para mulheres e idosos pois o risco de desnutrição aumentou em 2,4% para cada ano adicional de vida e em 0,4% para cada mês adicional de HD. Maior circunferência muscular do braço (CMB) e maior albumina sérica foram fatores de redução da desnutrição em 4,6% e 34,7%, respectivamente.

**Conclusão:**

Demonstrou-se que albumina sérica mais elevada e CMB preservada são bons indicadores de melhor estado nutricional. Maior MIS foi associado a pior qualidade de vida, idade mais avançada, menor renda e escolaridade, maior tempo em diálise e presença de comorbidades.

## Introdução

O número de pessoas com doença renal crônica (DRC) em hemodiálise (HD) de manutenção aumentou significativamente nas últimas décadas, principalmente devido ao envelhecimento da população e ao aumento de hipertensão e diabetes. Por outro lado, a terapia dialítica melhorou, aumentando a sobrevida dos pacientes em programas de diálise crônica^1,2^. O último censo da Sociedade Brasileira de Nefrologia mostrou um registro total de 153.831 pacientes em diálise^3^.

Embora as técnicas de diálise tenham avançado continuamente, a taxa de mortalidade entre os pacientes com DRC permanece elevada. O processo de desnutrição-inflamação é uma das principais causas de morbidade e mortalidade em pacientes com DRC. Esse achado é ilustrado pela associação inversa entre taxas de mortalidade e marcadores de estado nutricional adequado^4^. A perda proteico-energética (PEW, do inglês *protein-energy wasting*) é uma condição de desnutrição, inflamação, anorexia e perda de reservas corporais devido a condições inflamatórias e não inflamatórias em pacientes com DRC^5,6^.

A avaliação nutricional de indivíduos em HD continua sendo um desafio significativo, uma vez que não existe um método único capaz de diagnosticar com precisão o estado nutricional desses pacientes^7^. O Escore de Desnutrição-Inflamação (MIS, do inglês *Malnutrition-Inflammation Score*) é uma medida prática e reprodutível que pode ser usada tanto em pacientes em HD quanto em pacientes em diálise peritoneal. Estudos têm demonstrado que o MIS está associado a inflamação, anemia, qualidade de vida e mortalidade. Valores mais elevados de MIS estão independentemente associados a um risco maior de hospitalização^8,9^.

Embora a associação entre qualidade de vida (QV) e MIS na DRC tenha sido abordada em diversos trabalhos, há uma falta de estudos sobre essa associação em pacientes idosos. Deve-se ressaltar que a QV relacionada à saúde é um preditor forte e independente de hospitalização e óbito em pacientes submetidos à diálise^10^, e a avaliação regular da QV é recomendada pelo *Kidney Disease Outcomes Quality Initiative* (K/DOQI) como um dos parâmetros de adequação do tratamento^11,12^.

O objetivo deste estudo foi identificar o estado inflamatório e nutricional de pacientes idosos em hemodiálise e a associação entre estes fatores e sua qualidade de vida.

## Métodos

Este foi um estudo transversal realizado em três clínicas de hemodiálise na região metropolitana de Recife, no nordeste do Brasil. Os procedimentos estavam em conformidade com os padrões éticos do comitê responsável pela experimentação humana e com a Declaração de Helsinque de 1975, conforme revisada em 2013. O protocolo foi aprovado pelo Comitê de Ética (CAAE 64859716.5.0000.5207). Todos os participantes assinaram o termo de consentimento livre e esclarecido. A amostra foi composta por todos os pacientes das três clínicas de nefrologia, recrutados no período de março a dezembro de 2017, de ambos os sexos, com idade ≥ 60 anos, que haviam realizado hemodiálise três vezes por semana por pelo menos seis meses. Foram excluídos os pacientes idosos que apresentaram neoplasias, sorologia positiva para o vírus da imunodeficiência humana (HIV), hepatite viral, doença de Alzheimer, comprometimento cognitivo, membro amputado e/ou dependência de cadeira de rodas, doença de Parkinson e sequelas de AVC.

O protocolo de seleção de amostras está descrito na Figura 1. Inicialmente, foi realizado um teste de triagem para comprometimento cognitivo, o Mini Exame do Estado Mental (MEEM), para identificar quais idosos eram elegíveis para participar do estudo, com pontos de corte de 18 e 23, dependendo do nível de escolaridade dos participantes^13^.

**Figura 1:** Protocolo de seleção da amostra.

Os dados clínicos, sociodemográficos e de diálise foram coletados dos prontuários clínicos e os pacientes foram questionados.

Para avaliar a percepção do paciente sobre a QV, utilizou-se o questionário KDQOL-SF™^14^.

As medições antropométricas foram realizadas após a sessão de HD por três pesquisadores previamente treinados em ferramentas de monitoramento nutricional. O peso foi aferido em uma balança eletrônica Marte^®^. A estatura foi medida com um estadiômetro portátil Sanny^®^. Para o cálculo do índice de massa corporal (IMC)^15^, o peso atual (kg) foi dividido pela altura ao quadrado (m), com o resultado expresso em kg/m^2^.

A circunferência do braço (CB) foi medida no braço sem acesso vascular usando uma fita métrica inelástica Cescorf^®^. A medida foi registrada em centímetros^16^.

Para obter a circunferência muscular do braço (CMB), as medidas da dobra cutânea tricipital (DCT) no braço sem acesso vascular e da CB foram inseridas na equação de Frisancho^16^.

Para medir a DCT, foi utilizado um adipômetro Cescorf^®^. A adequação da CMB foi obtida por meio do valor do percentil 50 (Frisancho^17^) e calculada pela equação de Blackburn et al.^18^, na qual uma redução de >10% foi classificada como desnutrição^19^.

A circunferência da panturrilha (CP) foi avaliada na perna esquerda no ponto mais largo e classificada como desnutrição com uma CP <31 cm^20^.

Um teste de força de preensão manual (FPM) foi realizado na mão do paciente sem fístula arteriovenosa e foi obtido utilizando um dinamômetro de mão Saehan^®^ com ponto de corte para desnutrição de <23,3 kg, específico para a população em hemodiálise^21^.

A desnutrição-inflamação foi avaliada pelo MIS, conforme recomendado por Kalantar-Zadeh et al.^22^, e os pacientes foram classificados como nutridos quando obtiveram pontuação inferior a 6 e desnutridos com pontuação superior ou igual a 6^23^.

O KDQOL – SF^TM^, de acordo com a pontuação do MIS, inclui domínios específicos e genéricos. Os domínios específicos são Lista de Sintomas/Problemas, Efeitos da Doença Renal, Carga da Doença Renal, Papel Profissional, Função Cognitiva, Qualidade da Interação Social, Função Sexual, Sono, Apoio Social, Estímulo da Equipe de Diálise e Satisfação do Paciente. Os domínios genéricos são Funcionamento Físico, Função-Física, Dor, Saúde Geral, Bem-estar Emocional, Função-Emocional, Função Social, Energia/Fadiga^22^.

Os parâmetros laboratoriais foram coletados dos prontuários médicos de acordo com o acompanhamento de rotina do paciente, durante o qual uma amostra de sangue é coletada antes da sessão de hemodiálise. O estudo considerou os níveis de fósforo, cálcio, paratormônio, vitamina D, hemoglobina e albumina sérica.

Os dados foram analisados usando o SPSS. A descrição da amostra foi realizada por meio de frequências absolutas e relativas, médias e desvios padrão ou medianas e intervalos interquartis das variáveis avaliadas. Para a análise bivariada, foram utilizados o teste de associação Qui-quadrado, o teste t de Student ou o teste de Mann-Whitney. Nos casos em que as premissas do teste Qui-quadrado não foram atendidas, aplicou-se o teste exato de Fisher.

A análise multivariada incluiu fatores com uma significância de até 20% na análise bivariada. O modelo de Poisson com variância robusta foi aplicado para avaliar os fatores que aumentam o risco de desenvolver desnutrição nos pacientes idosos avaliados. Os fatores com nível de significância de 5% foram mantidos no modelo. Além disso, os intervalos de confiança para a razão de prevalência e o teste de Wald foram calculados para comparar os riscos entre os níveis dos fatores avaliados. Todas as conclusões foram tiradas com base em um nível de significância de 5%.

## Resultados

Este estudo incluiu 207 indivíduos, dos quais 131 eram do sexo masculino (63,3%), com idades entre 60 e 94 anos (68,23 ± 6,68). As características clínicas e nutricionais estão descritas na Tabela 1, com resultados estratificados pelo MIS como nutridos e desnutridos.

**Tabele 1:** Características clínicas e nutricionais de acordo com o escore de desnutrição-inflamação (MIS) de pacientes idosos em hemodiálise no nordeste do brasil, (n = 207)

		Escore de desnutrição-inflamação (MIS)		
Características	Total*	Nutrido (MIS < 6)*	Desnutrido (MIS = 6)*	Valor de p
	(n = 207)	(n = 99)	(n = 108)	
**Idade** (anos)**	68,23 ± 6,68	66,80 ± 4,96	69,55 ± 7,72	**0,002** ^1^
Sexo				
Feminino	76 (36,7)	24 (31,6)	52 (68,4)	**< 0,001** ^3^
Escolaridade				
< 9 anos (baixa escolaridade)	113 (54,6)	55 (48,7)	58 (51,3)	0,789^3^
Estado civil (com parceiro)				
Sem parceiro	73 (35,3)	28 (38,4)	45 (61,6)	**0,044** ^3^
**Renda familiar**				
< 1 salário mínimo	4 (1,9)	2 (50)	2 (50)	0,921^3^
1–5 salários mínimos	188 (90,8)	89 (47,3)	99 (52,7)	
> 5 salários mínimos	15 (7,2)	8 (53,3)	7(46,7)	
**Parâmetros Nutricionais**				
IMC (kg/m^2^)**	24,29 ± 4,21	25,22 ± 3,94	23,43 ± 4,28	**0,002** ^1^
CB (cm)**	28,10 ± 3,82	29 ± 3,32	27,29 ± 4,07	**0,001** ^1^
CMB (cm)**	23,82 ± 2,91	24,66 ± 2,47	23,04 ± 3,08	**< 0,001** ^1^
CP (cm)**	32,47 ± 3,38	33,43 ± 3,13	31,59 ± 3,38	**< 0,001** ^1^
FPM (kg)**	24,29 ± 8,73	27,10 ± 8,82	21,71 ± 7,85	**<0,001** ^1^
Albumina sérica (g/dL)**	3,8 ± 0,33	3,86 ± 0,30	3,74 ± 0,34	**0,008** ^1^
**Comorbidades**				
Diabetes mellitus	100 (48,3)	47 (47)	53 (53)	0,818^3^
Hipertensão arterial sistêmica	180 (87)	84 (46,7)	96 (53,3)	0,389^3^
Anemia	97 (46,9)	41 (42,3)	56 (57,7)	0,163^3^
**Parâmetros de Diálise**				
Dose de diálise (Kt/V)**	1,58 ± 0,36	1,52 ± 0,32	1,64 ± 0,38	**0,014** ^1^
Fósforo (mg/dL)**	4,84 ± 1,11	4,91 ± 1,08	4,77 ± 1,15	0,373^1^
Cálcio (mg/dL)**	8,87 ± 0,66	8,93 ± 0,68	8,82 ± 0,63	0,246^1^
Produto cálcio-fósforo (mg^2^/dL^2^)**	42,29 ± 10,89	43,27 ± 10,57	41,39 ± 11,14	0,214^1^
PTH (pg/ mL)***	311,30 (168; 493)	338,10 (173; 519)	298,25 (165,25; 460)	0,454^2^
Vitamina D (ng/mL)**	31,88 ± 12,35	33,20 ± 13,11	30,68 ± 11,53	0,144^1^
Hemoglobina (g/100 ml)**	10,98 ± 1,4	11,09 ± 1,45	10,87 ± 1,45	0,271^1^
Tempo em HD (meses)***	51 (22; 88)	43 (21; 78)	59 (24,50; 97,5)	**0,041** ^2^
Acesso vascular para HD				
*FAV ou prótese*	154 (74,4)	74 (48,1)	80 (51,9)	0,912^3^
*Permcath ou CDL*	53 (25,6)	25 (47,2)	28 (52,8)	
Duração da sessão de HD					
*3 a 3,5 horas*	8 (3,9)	4 (50)	4 (50)	1,000^4^
*4 horas*	199 (96,1)	99 (47,8)	108 (52,2)	

Abreviações: IMC, índice de massa corporal; CB, circunferência do braço; CMB, circunferência muscular do braço; CP, circunferência da panturrilha; FPM, força de preensão manual; PTH, paratormônio; HD, hemodiálise; FAV, fístula arteriovenosa; CDL, cateter duplo-lúmen.

Notas: *Frequência absoluta e relativa para variáveis categóricas;

**Média ± DP;

***Mediana e intervalo interquartil;

^1^Valor-p do teste t de Student;

^2^Valor p do teste de Mann-Whitney;

^3^Valor p do teste qui-quadrado;

^4^Valor p do teste exato de Fisher.

A prevalência de desnutrição-inflamação foi de 52,2% com base no MIS, e o escore médio de QV foi de 65,06 na população estudada, com base no KDQOL-SF^TM^.

O MIS foi associado à idade, ao sexo feminino e à ausência de parceiro. A maioria dos pacientes idosos tinha baixa escolaridade, renda mensal entre 1 e 5 salários mínimos, e sua terapia era financiada pelo Sistema Único de Saúde (SUS). No entanto, não houve diferença nessas características entre as categorias do MIS.

Em relação aos parâmetros nutricionais, as médias de IMC, CB, CMB, CP, FPM e albumina sérica foram significativamente menores nos pacientes classificados como desnutridos pelo MIS. Houve uma prevalência de desnutrição que variou de 54,1% segundo a FPM a 30,9% segundo a CP (Tabela 2). As comorbidades foram semelhantes entre os grupos.

**Tabela 2:** Prevalência de desnutrição de acordo com cada indicador nutricional em pacientes idosos em hemodiálise no nordeste do brasil (n = 207)

Indicador nutricional	Prevalência de desnutrição (%)
MIS (= 6)	52,2
Albumina sérica (< 3,8 g/dL)	38,2
CMB reduzida (> 10%)	39,1
FPM (< 23,3 kg)	54,1
CP (< 31 cm)	30,9

Abreviações: MIS, Escore de desnutrição-inflamação; CMB, circunferência muscular do braço; FPM, força de preensão manual; CP, circunferência da panturrilha.

Os parâmetros de diálise revelaram que a maioria dos idosos possuía uma fístula arteriovenosa (FAV)/prótese (74,4%) como acesso vascular para hemodiálise e realizava sessões de diálise de 4 horas (96,1%). A dose de diálise apresentou valores médios melhores no grupo de desnutrição (1,64 ± 0,38 versus 1,52 ± 0,32; p = 0,014). Os idosos mais afetados pelo processo de desnutrição-inflamação foram aqueles que estiveram em diálise por um período mais longo (43 versus 59 meses; p = 0,041).

A Tabela 3 descreve os 19 domínios do questionário KDQOL-SF^TM^. A maioria dos domínios apresentou escores mais elevados para os pacientes idosos nutridos. Nos domínios específicos Lista de Sintomas/Problemas (p < 0,001), Efeitos da Doença Renal (p = 0,004), Carga da Doença Renal (p = 0,004), Função Cognitiva (p = 0,001), Sono (p = 0,002), Apoio Social ao Paciente (p = 0,045) e Satisfação (p = 0,005), a QV dos pacientes desnutridos foi significativamente inferior à dos pacientes nutridos.

**Tabela 3:** Escores dos domínios do kdqol - sftm de acordo com o escore de desnutrição-inflamação (MIS) de pacientes idosos em hemodiálise no nordeste do brasil (n = 207)

		Escore de Desnutrição-Inflamação (MIS)		
Domínio avaliado	Total**	Nutrido (MIS < 6)**	Desnutrido (MIS = 6)**	Valor de p
Específicos	(n = 207)	(n = 99)	(n = 108)	
Lista de sintomas/problemas	79,22 ± 15,14	83,69 ± 13,62	75,13 ± 15,36	**< 0,001** ^1^
Efeitos da doença renal	69,38 ± 17,85	73,13 ± 16,40	65,94 ± 18,50	**0,004** ^1^
Carga da doença renal***	50 (37,5; 75)	50 (43,75; 75)	50 (25; 62,5)	**0,004** ^2^
Papel profissional***	0 (0; 50)	0 (0; 50)	0 (0; 50)	0,413^2^
Função Cognitiva	85,31 ± 17,08	89,56 ± 16,11	81,41 ± 17,07	**0,001** ^1^
Qualidade da Interação Social	80,97 ± 17,38	83,29 ± 17,85	78,82 ± 16,72	0,064^1^
Função Sexual*	86,17 ± 22,31	91,53 ± 15,60	75,78 ± 29,39	0,059^1^
Sono	69,24 ± 21,32	73,98 ± 20,39	64,88 ± 21,30	**0,002** ^1^
Apoio social	80,27 ± 28,23	84,34 ± 24,49	76,54 ± 30,91	**0,045** ^1^
Estímulo da equipe de diálise	72,89 ± 27,72	76,13 ± 26,06	69,90 ± 28,95	0,106^1^
Satisfação do Paciente	64,65 ± 22,46	69,19 ± 21,86	60,49 ± 22,27	**0,005** ^1^
**Genéricos (SF)**				
Funcionamento Físico***	45 (25; 75)	60 (35; 85)	35(20; 58,75)	**<0,001** ^2^
Função Física***	50 (25; 100)	75 (50; 100)	25 (0; 75)	**<0,001** ^2^
Dor***	77,50 (42,50; 100)	80 (45; 100)	75,0 (32; 50)	**0,004** ^2^
Saúde Geral***	50 (40; 70)	55 (45; 70)	50 (35; 65)	0,070^2^
Bem-estar emocional	72,37 ± 22,74	76,52 ± 23,27	68,55 ± 21,65	**0,011** ^1^
Função Emocional***	66,67 (0; 100)	100 (33,33; 100)	66,7 (0; 100)	**0,002** ^2^
Função Social	74,52 ± 23,94	80,05 ± 22,44	69,44 ± 24,24	**0,001** ^1^
Energia/Fadiga	62,08 ± 22,62	64,54 ± 23,86	59,81 ± 21,27	0,133^1^

Notas: *(n = 47);

**Média ± DP;

***Mediana e intervalo interquartil;

^1^Valor p do teste t de Student;

^2^Valor p do teste de Mann-Whitney. Nas dimensões do KDQOL - SF^TM^ o valor 0 reflete a QV mais baixa e o valor 100 reflete a QV mais elevada.

Os domínios genéricos da QV como Funcionamento físico, Função física, Dor, Bem-estar emocional, Função emocional e Função social também foram significativamente diferentes entre idosos nutridos e desnutridos.

A Tabela 4 apresenta o ajuste do modelo de Poisson para desnutrição e inflamação. Após a análise bivariada para avaliar a influência individual dos fatores no desenvolvimento de desnutrição e inflamação, a análise combinada resultou nos seguintes fatores significativos: sexo, idade, medida da CMB, nível de albumina sérica e tempo em HD. Observou-se também que o grupo feminino apresentou um risco 50,6% maior de desenvolver escores de desnutrição-inflamação mais elevados (piores) em comparação ao grupo masculino. Quanto à idade, observou-se que, com o aumento de um ano de vida, houve um aumento de 2,4% no risco de desenvolver escores mais elevados de desnutrição-inflamação. Para a CMB e a albumina, houve redução de 4,6% e 34,7%, respectivamente, no risco de desenvolver desnutrição-inflamação com aumento de 1 cm na CMB e de 1 g/dL na albumina. Quanto ao tempo em HD, para cada mês em que o paciente foi submetido ao tratamento, esse risco aumentou em 0,4%.

**Tabela 4:** Ajuste do modelo de poisson para escore de desnutrição-inflamação (mis) mais elevado

Fator avaliado	RP	IC95%	Valor de p
Sexo			
*Masculino*	1,000	–	–
*Feminino*	1,506	1,154 – 1,965	0,003
Idade	1,024	1,009 – 1,040	0,002
CMB	0,954	0,913 – 0,998	0,042
Albumina	0,653	0,452 – 0,943	0,023
Tempo em HD (meses)	1,004	1,002 – 1,006	<0,001

Abreviações: CMB, circunferência muscular do braço; HD, hemodiálise; RP, Razão de Prevalência; IC, Intervalo de Confiança.

## Discussão

A avaliação do estado nutricional de pacientes em HD é fundamental, uma vez que a desnutrição, especialmente a síndrome de PEW, é altamente prevalente e contribui para o aumento da morbidade e da mortalidade em pacientes em HD crônica^5,24^. O MIS é considerado um parâmetro sensível para avaliar a desnutrição e a inflamação em pacientes em HD e um preditor de mortalidade^22^, mas seu uso na população idosa tem sido pouco documentado.

De acordo com o MIS, a prevalência de desnutrição-inflamação na população estudada foi de 52,2%. Uma idade mais avançada foi associada a níveis mais elevados de MIS, resultado também encontrado em outros estudos^25^. O envelhecimento é um fator de risco para desnutrição, com um aumento de 2,4% no risco para cada ano de vida no modelo multivariado para essa população. Alterações fisiológicas ocorrem na terceira idade, resultantes da redução das necessidades e gastos energéticos, denominadas anorexia do envelhecimento. Essa anorexia fisiológica aumenta o risco de perda de peso e desnutrição quando um idoso desenvolve uma doença física ou psicológica^26^.

Para os idosos, a alimentação é sinônimo de família, união e qualidade de vida. O fato de os pacientes viverem com um parceiro e residirem com a família pode aumentar os cuidados domiciliares. Os pacientes idosos mais desnutridos foram aqueles que não tinham parceiro (61,6%). A DRC causa perdas funcionais que comprometem a independência e a autonomia, o que ocorre com mais frequência em pacientes idosos^27^.

As mulheres geralmente são menos prevalentes em estudos sobre DRC. Os verdadeiros efeitos protetores dos hormônios femininos na progressão da doença renal permanecem desconhecidos. Por outro lado, quando em diálise, as mulheres apresentam parâmetros clínicos piores, incluindo anemia, nutrição e qualidade de vida^28^. Nesse estudo, enquanto 36,7% da amostra eram mulheres, 68,4% estavam desnutridas e apresentaram um risco 50,6% maior de desenvolver pontuações MIS mais elevadas do que os homens.

Os parâmetros antropométricos e de diálise foram piores nos pacientes desnutridos, o que reforça a importância do ganho de peso corporal, especialmente de massa magra. Isso se deve ao fato de que o ganho de peso reflete em melhores condições funcionais e imunológicas, maior independência e menor morbidade e mortalidade relacionadas à desnutrição^29^.

A albumina sérica é um parâmetro nutricional importante em pacientes submetidos à terapia renal substitutiva, com um nível de 3,8 g/dL considerado normal. Os pacientes idosos deste estudo conseguiram atingir esse objetivo. Pereira et al.^30^ em um amplo estudo com 1.679 pacientes, investigaram o impacto da albumina na mortalidade após dois anos de hemodiálise e descobriram que a mortalidade foi significativamente maior no grupo com albumina abaixo de 3,8 g/dL. Szuck et al.^8^, que verificaram a capacidade dos indicadores nutricionais em predizer o risco de hospitalização em pacientes em hemodiálise, constataram que somente a albumina sérica foi capaz de predizer esse risco, e que os pacientes com valores inferiores a <3,8g/dL apresentaram uma incidência 2,47 vezes maior do que aqueles com níveis mais elevados de albumina.

Para a população deste estudo, a CMB foi um fator de proteção para a desnutrição-inflamação no modelo multivariado, pois com o aumento de um centímetro na CMB houve uma redução de 4,6% no risco de maior MIS.

A adequação da diálise avaliada pela medição das taxas de remoção de ureia, o Kt/V, indica quantas vezes a água corporal de um paciente é totalmente livre de ureia. A atual recomendação europeia é de um Kt/V igual ou superior a 1,2^31^. O Kt/V é muito importante na avaliação do estado nutricional, uma vez que a diálise inadequada resulta em estado urêmico, progredindo para náuseas, vômitos e anorexia, com consequente comprometimento da ingestão alimentar. Os resultados deste estudo demonstram que a diálise foi adequada para esses pacientes idosos, com Kt/V médio de 1,58. Os pacientes idosos desnutridos apresentaram os níveis mais elevados de Kt/V, demonstrando que a desnutrição pode ocorrer mesmo quando a diálise é eficiente. A desnutrição reduz o volume corporal, tornando os idosos mais suscetíveis a um aumento no Kt/V. A literatura indica que períodos mais longos em HD estão associados a um estado nutricional ruim e à inflamação^32^. Na amostra estudada, o risco de desnutrição-inflamação aumentou 0,4% para cada mês de terapia dialítica.

Com o aumento do acesso a novas terapias de diálise, como a hemodiafiltração de alto volume (HDF), e melhorias na qualidade da diálise, o estado nutricional de pacientes idosos em diálise crônica pode melhorar. No estudo de Maduell et al.^33^, os pacientes foram randomizados para diálise contínua ou HDF. Os resultados mostraram que a taxa de catabolismo proteico normalizada, que é um parâmetro do estado nutricional, foi maior nos pacientes randomizados para HDF.

A associação negativa entre condição nutricional e a perda proteico-energética (*Protein-energy wasting* (PEW) e a QV foi demonstrada e sua importância destacada por diversos estudos, uma vez que o tratamento dialítico, apesar de prolongar a sobrevida, tem um grande impacto em diferentes aspectos da vida dos pacientes^34–36^. Até o momento, não existe um método único, como um padrão ouro, capaz de diagnosticar a PEW, portanto, recomenda-se o uso de diferentes marcadores nutricionais^36^. É importante ressaltar que um indicador nutricional ideal deve ser capaz de prever os desfechos clínicos e identificar os pacientes que necessitam de intervenções nutricionais^37^. Apesar dos avanços na terapia de diálise, a PEW é comum nestas condições e está relacionada à inflamação, comorbidades associadas, estado hipercatabólico, redução da ingestão e anorexia^38^.

Este estudo observou uma associação negativa entre o MIS e diversos domínios do KDQOL-SF^TM^, sugerindo que a ocorrência de desnutrição-inflamação pode ter afetado negativamente a QV dos pacientes idosos nessa população em hemodiálise. Uma revisão sistemática demonstrou que idosos desnutridos tinham maior probabilidade de apresentar pior qualidade de vida (p <0,001; OR 2,85; IC 2,20–3,70). Os autores também observaram que, ao considerar o efeito do suporte nutricional em estudos de intervenção, há uma melhora significativa na QV desses indivíduos, tanto nos aspectos físicos quanto mentais^39^.

Com relação ao KDQOL-SF^TM^, o domínio que obteve a maior pontuação foi a função sexual, embora não tenha sido estatisticamente associado ao ao MIS. Esse domínio avalia se os pacientes se envolveram em atividades sexuais nas últimas quatro semanas e em que medida houve problemas com a excitação e a satisfação sexual. Esse resultado deve ser analisado cuidadosamente, já que parte da amostra relatou ter uma vida sexual ativa. No entanto, a disfunção erétil é muito comum em pacientes com insuficiência renal crônica devido a alterações hormonais, físicas, neurológicas e psicológicas^40^.

Outros domínios de pontuação elevada (melhor QV) associados ao MIS foram a função cognitiva e o apoio social. O domínio apoio social verifica o apoio recebido de familiares e amigos, e o domínio de qualidade da interação social avalia as relações familiares e sociais com o paciente. Esses aspectos podem ser muito valorizados pelos pacientes idosos, pois eles são altamente dependentes dos cuidados que a hemodiálise exige dos membros da família. É importante envolver a família no tratamento, incentivando sua participação e orientando-a a cooperar com a equipe de saúde^10^. Quando idosos em terapia renal substitutiva estão em um ambiente com relações familiares e sociais de apoio, eles geralmente aceitam o tratamento e se sentem motivados e gratos^41^.

O item função cognitiva avalia a percepção do participante sobre dificuldades de concentração e raciocínio, presença de confusão mental e atraso na reação a fenômenos que aconteceram ou foram mencionados. Pacientes com DRC correm risco de declínio cognitivo. O comprometimento cognitivo pode estar presente em qualquer estágio da doença renal e está associado a um risco aumentado de óbito e menor adesão ao tratamento. Embora os mecanismos que levam à perda da função cognitiva na DRC não estejam completamente claros, a literatura demonstra que condições clínicas, como a taxa de eliminação de toxinas urêmicas, podem induzir lesões neuronais^41^. Oliveira et al.^10^ relataram uma correlação inversa significativa entre QV e número de tratamentos perdidos, revelando que quanto menor a QV nesse aspecto, maior o número de tratamentos perdidos, representando assim uma menor adesão ao tratamento.

O sono foi um dos domínios com menor pontuação, e a desnutrição-inflamação esteve significativamente associada, uma vez que, nessa população, os pacientes nutridos dormiam melhor do que os desnutridos. A má qualidade do sono não é incomum em pacientes em hemodiálise, com uma prevalência que varia de 41% a 83%. É mais comumente associada a mulheres, idade avançada, presença de depressão, doença cardiovascular, má qualidade da terapia de diálise e comprometimento da qualidade de vida relacionada à saúde^42^.

O baixo escore de QV como efeito da doença renal demonstra como as limitações da diálise, tais como ingestão de líquidos e dieta restritas, dificuldades de locomoção, dependência da equipe de saúde, aparência pessoal e outras limitações, são um inconveniente para os pacientes idosos. A baixa QV reflete a interferência que a doença renal causa na vida desses pacientes, principalmente devido ao tempo que eles passam em tratamento^41^. Os resultados observados para a pontuação do papel profissional estão relacionados ao fato de que quase todos os idosos não estão mais em idade produtiva e os que ainda estão trabalhando no início da terapia de diálise frequentemente se aposentam.

No que diz respeito aos domínios genéricos (SF-36), os pacientes idosos deste estudo apresentaram escores mais elevados nos domínios que envolvem aspectos mentais e escores mais baixos nos domínios que envolvem aspectos físicos. Com o processo de envelhecimento, as limitações causadas pelo tratamento de hemodiálise tendem a aumentar, especialmente as limitações físicas. No entanto, o lado emocional se torna mais forte com o avanço da idade. Os idosos são psicologicamente mais capazes de lidar com as demandas do tratamento, suas expectativas são mais realistas e eles são mais capazes de adaptar-se ao seu estado de saúde^10,32^.

As limitações do estudo estão relacionadas ao desenho transversal, no qual o grupo foi avaliado somente uma vez. Portanto, não é possível estabelecer relações causais, ou seja, se a desnutrição-inflamação causa comprometimento da QV ou se uma QV comprometida causa redução do apetite e, portanto, desnutrição. Estudos transversais com pessoas idosas estão sujeitos ao viés de sobrevivência, pois os participantes mais graves podem ter falecido, ou seja, os resultados observados foram de uma população de idosos sobreviventes. Além disso, a falta de análise da perda de massa livre de gordura e da ingestão alimentar impossibilitou uma avaliação mais precisa da nutrição em idosos em hemodiálise.

Em resumo, os resultados mostraram que o escore de desnutrição-inflamação, MIS, foi associado a uma pior QV para pacientes idosos em hemodiálise. A albumina sérica e a CMB foram consideradas fatores de proteção, evitando elevação do MIS. Além disso, pessoas de idade mais avançada, com mais tempo de terapia dialítica e mulheres, apresentam um risco maior de desenvolver desnutrição-inflamação.

## Data Availability

Os dados subjacentes a este artigo estão disponíveis no artigo e em seu material suplementar on-line.
